# Somatostatin and Somatostatin-Containing Neurons in Shaping Neuronal Activity and Plasticity

**DOI:** 10.3389/fncir.2016.00048

**Published:** 2016-06-30

**Authors:** Monika Liguz-Lecznar, Joanna Urban-Ciecko, Malgorzata Kossut

**Affiliations:** ^1^Department of Molecular and Cellular Neurobiology, Nencki Institute of Experimental BiologyWarsaw, Poland; ^2^Department of Biological Sciences and Center for the Neural Basis of Cognition, Carnegie Mellon UniversityPittsburgh, PA, USA; ^3^Department of Psychology, University of Social Sciences and Humanities (SWPS)Warsaw, Poland

**Keywords:** somatostatin, interneurons, plasticity, GABA, SOM, inhibition

## Abstract

Since its discovery over four decades ago, somatostatin (SOM) receives growing scientific and clinical interest. Being localized in the nervous system in a subset of interneurons somatostatin acts as a neurotransmitter or neuromodulator and its role in the fine-tuning of neuronal activity and involvement in synaptic plasticity and memory formation are widely recognized in the recent literature. Combining transgenic animals with electrophysiological, anatomical and molecular methods allowed to characterize several subpopulations of somatostatin-containing interneurons possessing specific anatomical and physiological features engaged in controlling the output of cortical excitatory neurons. Special characteristic and connectivity of somatostatin-containing neurons set them up as significant players in shaping activity and plasticity of the nervous system. However, somatostatin is not just a marker of particular interneuronal subpopulation. Somatostatin itself acts pre- and postsynaptically, modulating excitability and neuronal responses. In the present review, we combine the knowledge regarding somatostatin and somatostatin-containing interneurons, trying to incorporate it into the current view concerning the role of the somatostatinergic system in cortical plasticity.

## Introduction

Over forty years ago, scientists discovered a small 14 amino-acid-long peptide that was able to inhibit the release of growth hormone from the hypothalamus. They called it somatotropin release inhibiting factor (SIRF) or somatostatin (SOM or SST; Krulich et al., 1968; Brazeau et al., 1973). It was quickly established that SOM is present also beyond hypothalamus, across many regions of central and peripheral nervous system, but also in non-neuronal tissues, like gastrointestinal tract and endocrine pancreas or thyroid. Later, a number of additional SOM synthesis sites have been identified and included the placenta, kidney, retina and cells of the immune system (Patel, 1999).

Generally, the function of SOM is to inhibit the release of several biologically active substances, like: growth hormone, insulin, glucagon, gastrin, secretin, cholecystokinin (Thorner et al., 1990; Herzig et al., 1994; Lloyd et al., 1997; Yao et al., 2005; Rutter, 2009; Chey and Chang, 2014; respectively). However, in the nervous system it is localized in considerable proportion of GABAergic neurons acting as neurotransmitter or neuromodulator (Reichlin, 1995). In the scientific reports somatostatin is often treated just as a marker for particular GABAergic interneuronal subpopulation. However, somatostatin itself acts pre- and postsynaptically, modulating excitability and neuronal responses. Special characteristics and connectivity of somatostatin-containing neurons set this neuronal subpopulation up as a significant player in shaping activity and plasticity of the nervous system. In this review we combine the knowledge regarding somatostatin and somatostatin-containing interneurons, trying to incorporate it into the present view concerning the role of somatostatinergic system in cortical plasticity.

## Somatostatin

Somatostatin, as many other hormones, is synthesized as a part of larger prohormone-preprosomatostatin, and then undergoes proteolytic cleavage to produce one of two active forms referring to as SS-14 and SS-28, which reflects their amino acid chain length (Schally and Meyers, 1980). Which and in what amount a particular form is secreted depends on the tissue, since both forms have different biological potency for inhibition of different substances. For example, SS-14 is predominant in the nervous system, whereas SS-28 is more biologically active in the endocrine pancreas. The longer form SS-28 can be further processed to SS-14 (Schally and Meyers, 1980).

Physiological action of somatostatin is mediated by a family of structurally related proteins which have different pharmacological properties and distinct pattern of expression in the central nervous system and peripheral tissues (Bruno et al., 1992). Somatostatin receptors, known as SSTR1 to SSTR5 (Patel et al., 1994) were cloned in the early 1990’s and they all belong to a family of G protein-coupled receptors. As all the G-coupled receptors, they have seven transmembrane domains and are encoded by separate genes segregated on different chromosomes.

The localization and distribution of SOM receptors in the CNS and periphery depend on tissue and species. They are widely distributed in many tissues, with distinct but overlapping expression pattern, often as multiple subtypes coexisting in the same cell (Kossut et al., 1989). The five receptors share common metabotropic signaling pathways, such as inhibition of adenylyl cyclase, activation of phosphotyrosine phosphatase, and modulation of mitogen-activated protein kinase (MAPK). Some of the subtypes are also coupled to voltage-dependent Ca^2+^ channels (SSTR1, 2), inward rectifying K^+^ channels (SSTR2, 3, 4, 5), Na^+^/H^+^ exchanger (SSTR1), phospholipase C (SSTR2, 5), phospholipase A_2_ (SSTR4), AMPA/kainate glutamate channels (SSTR1, 2; Patel, 1999; Table 1).

**Table 1:** **Properties and distribution of somatostatin (SOM) receptors**.

	SSTR1	SSTR2	SSTR3	SSTR4	SSTR5
**Affinity for SS14 and SS28**	SS14 = SS28	SS14 = SS28	SS14 = SS28	SS14 = SS28	SS14 < SS28
**Synaptic localization**	Mainly presynaptic	Postsynaptic	Extrasynaptic (neuronal cilia)	Postsynaptic	postsynaptic
**Transducer**	G_i_/G_o_ family	G_i_/G_o_ family G_q_/G_11_ family G protein independent mechanism	G_i_/G_o_ family G_q_/G_11_ family	G_i_/G_o_ family	G_i_/G_o_ family G_q_/G_11_ family
**Effectors**	AC; PTP; PLC NHE1 AMPA/kainate CA^2+^ channels K^+^ channels	AC; PTP; PLC PLD; MAPK AMPA/kainate CA^2+^ channels K^+^ channels	AC; PTP; PLC MAPK, NHE1 K^+^ channels	AC; PTP; PLC MAPK; NHE1 PLA CA^2+^ channels K^+^ channels	AC; PTP; PLC MAPK; CA^2+^ channels K^+^ channels
**Distribution in the Brain**	**High level**: amygdala cortex hippocampus hypothalamus **Medium level**: cerebellum midbrain spinal cord striatum thalamus	**High level**: amygdala, cortex, hippocampus hypothalamus **Medium level**: cerebellum midbrain striatum spinal cord thalamus	**High level**: amygdala cerebellum cortex hippocampus, olfactory bulb striatum **Medium level**: hypothalamus midbrain preoptic area thalamus	**Medium level**: amygdala cerebellum cortex hippocampus olfactory bulb preoptic area	**High level**: hypothalamus preoptic area **Medium level**: amygdala cerebellum cortex hippocampus striatum

*Abbreviations: AC - adenylate cyclase; MAPK - mitogen-activated protein kinases; NHE1 - Na^+^/H^+^ exchanger; PLA - phospholipase A; PLC - phospholipase C; PLD - phospholipase D; PTP - protein tyrosine phosphatase. Reference: Moller et al. (2003) and Alexander et al. (2015)*.

Although different somatostatin receptor subtypes SST1-SST5 show overlapping distributions, they have also a high degree of specialization with regard to their subcellular targeting. While SSTR2 and SSTR4 mediate mainly postsynaptic responses, SSTR1 is poised to modulate presynaptic responses. In contrast, the SSTR3 appears to be excluded from classical synaptic localization and is selectively targeted to neuronal cilia (Schulz et al., 2000). All five receptors SSTR1–5 have been shown to be expressed in many regions of the fetal rodent brain with SSTR2 being predominant (Bologna and Leroux, 2000). In postnatal rat brain particular receptors achieve their peak expression sequentially: SSTR1 in P4-P7; SSTR3 and 5 in P7-P14 and SSTR4 around P21. In adult cortex SSTR1 and SSTR2 dominate, each exhibiting a particular distribution pattern across the cortical layers (Bologna and Leroux, 2000).

Among the biological effects of SOM are hindering of secretion, achieved by inhibition of exocytosis through decreasing cAMP production; induction of cell cycle arrest via modulation of MAPK (SSTR1, 2, 4, and 5); triggering apoptosis by activation of p53 and the pro-apoptotic protein Bax (SSTR3). Some biological responses display a selectivity for particular receptor subtype: growth hormone secretion regulation (SSTR2 and 5), insulin secretion (SSTR5), glucagon secretion (SSTR2), and immune responses (SSTR2; Patel, 1999; Table 1).

In CNS, somatostatin functions as a neurotransmitter or neuromodulator with mainly inhibitory action. It is also an important regulator of cell proliferation and differentiation (Viollet et al., 2008). In the cerebral cortex, somatostatin is a co-transmitter of GABAergic inhibitory neurons. As a co-transmitter it can modulate the activity of the surrounding neurons. Neuropeptides which are co-transmitters differ from classical neurotransmitters in size and mechanism of action. They act slower than the classical neurotransmitters and are involved in supporting a fine-tuning of neuronal signaling. It was shown that GABAergic interneurons which express neuropeptides are targeted by catecholaminergic and serotoninergic afferents with different preferences, suggesting their role in modulation of emotional and cognitive processes. Somatostatin-containing neurons are innervated by noradrenergic and serotoninergic fibers (Paspalas and Papadopoulos, 1999, 2001), but they are also affected by other neuromodulators, especially acetylcholine, which acting via intracortical pathways directly facilitates their activity (Chen et al., 2015). However, acetylcholine can also regulate the activity of SOM neurons indirectly, through basal forebrain cholinergic activation of VIP-containing interneurons, which contact and inhibit SOM interneurons (Letzkus et al., 2011; Jackson et al., 2016).

### Control of SOM Release

Early observations by Gamse et al. (1980), revealed that somatostatin is released from cultured hypothalamic cells even in the absence of exogenous stimuli, in calcium- dependent manner. Tapia-Arancibia and Astier (1989) showed that increase of SOM release is achieved by membrane depolarization. Fontana et al. (1996) have shown that in hippocampus, glutamate can stimulate somatostatin release through the activation of ionotropic NMDA and AMPA receptors; furthermore, experiments by Rage et al. (1994) revealed that in primary cultures of hypothalamic neurons release of somatostatin was elicited by NMDA application.

GABA was shown to inhibit spontaneous release of SOM (Gamse et al., 1980). This result was further specified by Llorens-cortes et al. (1992) who, by stimulating GABAA receptors *in vivo* with muscimol and diazepam, have shown that it decreased SOM content in mouse hypothalamus and cortex.

### Effect of SOM Release

Somatostatin, like other neuropeptides, is stored in dense-core vesicles residing away from the active zone in contrast to the classical neurotransmitters localized in small synaptic vesicles within the active zone. Dense-core vesicles require repetitive action potentials at high frequencies to release neuropeptides and due to large size neuropeptides diffuse through the fusion pore slower than classical neurotransmitters. Moreover, the receptors for neuropeptides are often in some distance from the release site and there are no selective reuptake mechanisms. Thus, somatostatin acts slower and its effects are longer lasting when compared with classical neurotransmitters (Baraban and Tallent, 2004).

Somatostatin can exert its modulatory effect pre- or postsynaptically. However, results concerning the direction and mechanisms of somatostatin effect on synaptic transmission are conflicting, although most studies reported its inhibitory effect. Many of them showed that SOM inhibits excitatory synaptic transmission and neuronal excitability showing silencing or hyperpolarizing effect of somatostatin in hypothalamus, spinal cord, hippocampus or cerebral cortex (Pittman and Siggins, 1981; Grilli et al., 2004). Somatostatin is also able to decrease GABA release, as was shown in thalamus, forebrain and striatum (Leresche et al., 2000; Momiyama and Zaborszky, 2006; Lopez-Huerta et al., 2008). Moreover, an activity-dependent release of SOM in hippocampal cultures resulted not only in reduction of mEPSCs frequency, but also in the number of dendritic spines and in the density of pre- and postsynaptic markers of excitatory synapses (Vglut-1 and GluR2). Thus, beside modulation of neuronal activity, SOM can also regulate the morphology and function of excitatory synapses (Hou and Yu, 2013).

However, there were also studies reporting excitatory effects of SOM (Olpe et al., 1980; Delfs and Dichter, 1983). Also, facilitatory effect of SOM on the generation of LTP in hippocampus via modulation of muscarinic cholinergic receptors was shown by Nakata et al. (1996). Gardette et al. (1995) showed in dissociated hypothalamic neurons that somatostatin was able to increase or decrease glutamatergic responses of developing neurons and these effects changed with time. Thus, somatostatin could modulate glutamate sensitivity of hypothalamic neurons with either synergistic or antagonistic effect, which was dependent on the activated receptor subtype. On the other hand, Delfs and Dichter (1983) showed, that the effect of SOM, applied to cortical neurons in culture, can be dose dependent with smaller dose inducing excitation and higher dose being inhibitory. Mancillas et al. (1986) using single-unit recordings in hippocampus and parietal cortex have noticed that when applied alone, somatostatin had inhibitory effect on spontaneous firing of pyramidal neurons, but if applied together with small amounts of acetylcholine it caused a dose dependent enhancement of acetylcholine-induced facilitation. In summary, acting presynaptically, somatostatin is able to decrease neurotransmitter release, which would diminish the input on the principal neurons, while postsynaptic action of SOM results most often in hyperpolarization of the target neuron, inducing a slow but long-lasting inhibition. However, acting synergistically with other neurotransmitters, somatostatin can reverse the direction of its effect suggesting a complexity of possible effects of somatostatin at a cellular level.

### Development

Cumulative evidence suggests that in brain development, somatostatin itself, plays a role as a trophic or apoptotic factor, influencing synaptogenesis, proliferation of cerebellar neuroblasts and axonal pathfinding (Gonzalez et al., 1992; Ferriero et al., 1994). Somatostatin-containing neurons, participate in regulation of infragranular cortex assembly and functional maturation (Tuncdemir et al., 2016).

During development, interneurons incorporate into the six-layered neocortex in an inside-out fashion. In rodents, majority of cortical interneurons is produced in the ventral telencephalon in the medial, lateral and caudal ganglionic eminences (Marin et al., 2001) as well as in the preoptic area (Gelman et al., 2009) and then they tangentially migrate into developing cortex. Already at these prenatal stages, pyramidal neurons are integrated into transient GABAergic networks, which then mature gradually during the first postnatal weeks (Luhmann et al., 2014). The prevalent early-born cortical interneuron populations include SOM and parvalbumin (PV) interneurons (Tuncdemir et al., 2016). It was discovered that distinct ganglionic eminences give rise to phenotypically different subgroups of cortical interneurons and so medial ganglionic eminence (MGE) produces approximately 70% of neocortical interneurons with SOM interneurons being produced in dorsal MGE and PV interneurons in ventral MGE (Xu et al., 2004; Rudy et al., 2011; Le Magueresse and Monyer, 2013). However, despite their common embryonic origin, SOM neuronal progenitors represent the earliest interneuronal population migrating to deep layers of cortical plate as early as E17.5 (Miyoshi and Fishell, 2011), while development of PV neurons innervation extends into later postnatal stages (Daw et al., 2007). Early-born SOM neurons localize mainly in 5/6 cortical layer and often persist throughout development. They receive dense transient thalamocortical innervation and provide an input to the excitatory neurons and inhibitory PV-containing neurons during the first postnatal week. Later on, they develop into typical adult-like L5/6 SOM interneurons (Tuncdemir et al., 2016).

Hogan and Berman (1993) observed postnatal development of somatostatinergic neurons in visual cortex of cat. They have shown that at 1 week of age, SOM-IR neurons were only found in deep layers of the developing cortex. By 2 weeks of age, SOM-IR neurons were found in layer 4 (L4) and 1 week later, they were located in all layers of the cortex except L1. Similar developmental pattern was seen by Bendotti et al. (1990) for preprosomatostatin mRNA in mouse cerebral cortex and by Papadopoulos et al. (1993) for protein in visual areas of rat. SOM neurons were visible from first postnatal week, then appeared to increase in numbers up to about 3 weeks and thereafter decline dramatically to adult levels, which were 14–19% of the peak levels.

In neonatal rat brain SOM cells were detected even at P0 and were located in region of subcortical plate, but from the end of the first postnatal week they were visible across all cortical layers. Their number increased significantly up to the 3–4th postnatal week (Lee et al., 1998). Other studies showed that SOM is expressed by some interneuron progenitors in the cerebral murine cortex and in migrating populations in the ventrolateral cortex at birth (Mukhopadhyay et al., 2009). On the other hand, Ouellet and de Villers-Sidani (2014) showed somatostatin expression in rat auditory cortex not earlier than between P9 and P20.

### Distribution

In adult animals, somatostatin is widely distributed in the whole rodent brain except the cerebellum. Dense population of somatostatin-containing cell bodies were found in neocortex, hippocampus, amygdala, piriform cortex, nucleus caudatus, nucleus accumbens, hypothalamus, striatum, olfactory regions and the brainstem (Epelbaum et al., 1994; Tomioka et al., 2005). Within neocortex, there are two main types of somatostatin-containing neurons: Martinotti cells reaching terminal branches of the apical dendrites of neocortical pyramidal cells in L1 and nest and small basket cells with axonal ramification limited to one layer (Druga, 2009). Martinotti cells are localized in superficial (L2/3) and deep (L5/6) cortical layers (Markram et al., 2004; Wang et al., 2004; Ma et al., 2006) whereas small basket cells can be found in all cortical layers, but majority is localized in L4 of cerebral cortex (Kawaguchi and Kubota, 1997; Markram et al., 2004; Ma et al., 2006; Viollet et al., 2008).

## SOM-Containing Neurons in Cerebral Cortex

In cerebral cortex, somatostatin is usually co-localized with gamma-aminobutyric acid (GABA), the main inhibitory neurotransmitter. Some recent articles claim almost complete colocalization (Kubota et al., 1994; Uematsu et al., 2008). Others however, report some proportion of SOM neurons which seems not to express GABAergic markers and there is no consistency how big this population can be. Some authors reported relatively small number of SOM+/GABA− cells, amounting to 3–20% in rat hippocampus and cortex, (Kosaka et al., 1988; Gonchar et al., 2007), while in other observations this group was much bigger: −40 to 70% in the entorhinal cortex and amygdala (Wouterlood and Pothuizen, 2000; McDonald and Zaric, 2015). However, since those estimations were made on the basis of immunostaining co-localization of different GABAergic markers and SOM, there is a possibility of underestimation if some of them is below the detection threshold.

Inhibitory (GABAergic) neurons, although comprise only 10–20% of cortical neurons, form very heterogeneous group concerning their chemistry, morphology, electrical properties and synaptic connectivity. They not only control the overall cortical activity level, but also determine the timing of action potential firing and regulate the postnatal development of neuronal circuitry (Markram et al., 2004).

The specific functions of cortical GABAergic interneurons are accomplished through an astonishing diversity of subgroups which can be distinguished using different determinants: somatodendritic morphology, chemical and genetic markers, functional properties and connectivity (Kubota and Kawaguchi, 1994; Kawaguchi and Kubota, 1997; Ascoli et al., 2008). One of such categorization can be made on the basis of the expression of other molecules (neuropeptides, calcium binding proteins (CBPs), neuronal nitric oxide synthase (nNOS); Kubota et al., 1994, 2011; Uematsu et al., 2008; Kubota, 2014). Since these markers are often closely correlated with morphology or physiology of neuronal group in which they are expressed, they can serve as a tool for characterization and classification. Commonly used markers for classification of GABAergic interneurons are PV, SOM, calretinin (CR), calbindin (CB), vasoactive intestinal peptide (VIP), 5HT3a receptor, substance P receptor, neuropeptide Y (NPY), cholecystokinin and cholin acetyltransferase (Kubota et al., 1994, 2011; Markram et al., 2004; Xu et al., 2004; Ascoli et al., 2008; Rudy et al., 2011).

In the neocortex, somatostatin containing group of interneurons constitutes about 20–30% of GABAergic neurons, being the second largest subpopulation after PV expressing neurons (40–50%; Uematsu et al., 2008; Rudy et al., 2011). Those two groups are nonoverlapping, in frontal, somatosensory and visual cortex of mouse (Xu et al., 2010) as well as in rat visual cortex. However, there are reports that clearly show colocalization of SOM and PV in mouse and rat hippocampus (Jinno and Kosaka, 2000), mouse subiculum and olfactory bulb (Lepousez et al., 2010; Nassar et al., 2015). About 20% of SOM neurons co-express other markers of GABAregic interneurons: CB, CR, NPY, substance P receptors or nNOS (Gonchar et al., 2002; Markram et al., 2004; Endo et al., 2016).

Overall, SOM positive neurons comprise 1–3% of neocortical neurons and they are a heterogeneous group with different expression of CBPs and diverse electrophysiological characteristics. In this group mostly multipolar, but also bipolar and fusiform neurons can be found. Somatostatin expression was found in Martinotti cells, in a limited fraction of small and nest basket cells and has also been detected in some double-bouquet and bi-tufted cells (Wang et al., 2004; Ma et al., 2006). Although somatostatin cells are called dendrite-preferring interneurons, since they innervate mainly shafts and spines of basal and apical dendrites of pyramidal neurons, their axons can also make axo-somatic as well as axo-axonic synapses (Gonchar et al., 2002).

Despite the fact that interneurons are usually short-range neurons, providing local connections, several investigations delivered evidence that in many species (monkeys, rodents, carnivores) a small number of inhibitory neurons establish long-range cortico-cortical connections (Tomioka et al., 2005). They connect different cortical areas both ipsilaterally and contralaterally and are involved in synchronization of rhythmic activity between distant cortical areas, serving to coordinate large-scale network activity (Melzer et al., 2012; Caputi et al., 2013). Most of them are classified as somatostatin, nNOS and NPY containing GABAergic neurons (Tomioka et al., 2005; Higo et al., 2007; McDonald and Zaric, 2015). Tomioka et al. (2005) found also that cortico-striatal GABAergic projection is constituted mainly by somatostatin positive, nNOS negative neurons which may be neurochemically distinct from cortico-cortical group. Considering that both cortico-cortical and cortico-striatal GABAergic projection neurons are subpopulations of somatostatinergic cortical neurons, it is likely that their chemical, morphological and electrical properties are characteristic for unique neuronal networks, to which they belong.

## SOM in Brain Pathology

There is robust evidence that alterations in somatostatin level, somatostatin-containing neurons and SOM receptors are associated with many pathological conditions like epilepsy, neuropsychiatric and neurodegenerative disorders (Lin and Sibille, 2013).

Epilepsy is one of the most common neurological disorders characterized by the occurrence of recurrent, unprovoked seizures caused by an alteration of the subtle balance between excitation and inhibition in the brain. It is unclear if distinct types of interneurons are selectively involved in the generation of epileptiform activity, but SOM interneurons are susceptible to seizures-induced death and a decline of SOM containing neurons is regarded as a hallmark of epileptic hippocampus (Clynen et al., 2014). Loss of SOM neurons has been confirmed in virtually all models of acquired epilepsy, including kindling, status epilepticus and traumatic brain injury models (Houser, 2014). There are data showing that application of SOM or its receptor SSTR2 agonist reduced the severity and duration of seizures, while somatostatin antiserum had proepileptic effects (Tallent and Qiu, 2008).

Decreased level of SOM in cerebro-spinal fluid (CSF) of patients with major depressive disorder was confirmed in several studies and examination of human post-mortem brains revealed region-specific somatostatin deficits in prefrontal and anterior cingulate cortex as well as in amygdala (Tripp et al., 2011; Lin and Sibille, 2013).

Alterations in somatostatinergic system were reported also in other neuropsychiatric disorders. In schizophrenia a reduction of CSF somatostatin, decreased *SOM* gene expression in prefrontal cortex and reduced density of SOM^+^ neurons in the entorhinal cortex and hippocampus was shown Benes (2015). In bipolar disorder, which characterizes with mood fluctuation, an increased CSF SOM was observed during manic periods while a decline of SOM interneurons was reported in hippocampus and entorhinal cortex (Konradi et al., 2011). Thus, changes of SOM cerebrospinal fluid level are state-dependent and seem not to be specific for any particular disorder.

Alzheimers disease (AD) is one of the most common neurodegenerative disease attacking the brain and leading to dementia. It is characterized by the presence of two kinds of microscopic lesions called senile plaques and neurofibrillary tangles. Davies et al. (1980) have shown that in AD patients concentration of SOM in cortex and hippocampus was lower than in healthy subjects. Later it was shown that the SOM deficiency correlated with the illness severity and cognitive deficits (Epelbaum et al., 2009). Saito et al. (2005) identified SOM as a positive modulator of activity of neprilysin- an enzyme involved in Aβ degradation. He suggested, that decrease of SOM expression can act as a trigger for Aβ accumulation contributing to late-onset sporadic AD. However, Dournaud et al. (1995), did not find close relationship between somatostatin deficit and neuropathology of AD and Martel et al. (2015) showed an enrichment of SOM neurons and fibers in olfactory peduncle and cortex of human postmortem brains.

Thus, some authors indicate alterations of SOM as a strong marker of AD pathology others however, suggest that considering interaction of cholinergic pathways with SOM interneurons, the changes observed in somatostatinergic system in AD might be secondary to the degeneration of cholinergic afferents from the nucleus basalis, which possess SOM receptors.

Indisputable, reduction of cortical SOM concentrations is not restricted to AD but is associated also with other neurodegenerative diseases related to cognitive dysfunctions, including Parkinson’s disease (Jiménez-Jiménez et al., 2014) and multiple sclerosis (Roca et al., 1999). Taken together, the presented results suggest that somatostatin alterations are common features of many neurological disorders and diseases, thus its direct involvement in induction of particular neuropathology is still to be confirmed.

## Electrophysiological Properties of SOM Interneurons

SOM neurons display diverse spiking responses to somatic current injections in patch-clamp recordings. It should be mentioned that, the same firing phenotype has variety of names in different studies (Kawaguchi and Kubota, 1996, 1997; Gibson et al., 1999; Beierlein et al., 2003; Goldberg et al., 2004; Wang et al., 2004; Ma et al., 2006).

In the neocortex, the majority of SOM neurons displays so called classical accommodating (c-AC; Wang et al., 2004) spiking responses to current injection which might be analogous to other terms such as regular spiking (RS) non-pyramidal (RSNP, Kawaguchi and Kubota, 1996, 1997) or low-threshold spiking (LTS, Kawaguchi, 1993; Gibson et al., 1999; Goldberg et al., 2004) in other studies.

“Classic” LTS interneurons generate rebound spike bursts following somatic hyperpolarization (Kawaguchi and Kubota, 1996; Goldberg et al., 2004) and not every SOM cell shows this phenomenon (Goldberg et al., 2004; Ma et al., 2006). Additionally, in response to hyperpolarizing currents, LTS cells display a sag which is mediated by the hyperpolarization-activated cationic current *I*h (Ma et al., 2006).

The most characteristic feature of LTS cells that distinguishes them from RS excitatory neurons and fast spiking (FS) interneurons is the shape of the afterhyperpolarization (AHPs), which consists of two components with early and late peaks (Kawaguchi, 1993; Kawaguchi and Kubota, 1996; Beierlein et al., 2003; Ma et al., 2006), also referred as a triphasic waveform by Ma et al. (2006). The spike-width of SOM cells is intermediate: broader than in PV cells and narrower than in excitatory neurons (Ji et al., 2016). In addition, SOM neurons expressing CR have slightly broader action potentials with slower AHPs than SOM/CR (Xu et al., 2006).

There is also a small subset of SOM neurons (15%, Wang et al., 2004) responding with a spike burst at the beginning of the discharge which is called burst-accommodating (b-AC, Wang et al., 2004) or burst spiking non-pyramidal cell (BSNP, Kawaguchi and Kubota, 1996).

Moreover, a small fraction of SOM cells (8%, Wang et al., 2004) shows non-accommodating firing response (NAC) which is analogous to FS responses characteristic to PV interneurons. Lastly, the minority of SOM neurons also shows burst irregular spiking responses (b-IS, Wang et al., 2004) or stuttering responses (STUT, Ma et al., 2006).

It is worthwhile to mention that VIP interneurons display similar LTS/RSNP/BSNP firing phenotype (Kawaguchi and Kubota, 1997). For this reason, LTS is not a definitive determinant of SOM neurons and careful consideration should be given to studies using the firing phenotype as the only one category to determine a subset of interneurons since this population might include interneurons expressing different molecular markers.

Due to diversity in the firing pattern and also morphology of SOM cells, considerably studies should combine electrophysiological, morphological and molecular approaches to reveal potential diversity within SOM interneuron population.

Generally, SOM neurons in L2/3 and L5 share similar electrophysiological properties whereas L4 SOM neurons are distinctive (Ma et al., 2006). SOM neurons located in L2/3 and L5 have a very high input resistance and more depolarized resting membrane potentials in comparison to FS interneurons, whereas L4 SOM neurons have these membrane properties comparable to FS (Ma et al., 2006). L5 SOM neurons respond more frequently with the initial burst than SOM neurons in other cortical layers and many L4 SOM neurons fire in stuttering or FS-like pattern (Ma et al., 2006). Using SOM-Cre-mouse line (Taniguchi et al., 2011), in which Cre recombinase is expressed in SOM-containing neurons, Hu et al. (2013) found, that a small portion (6–10%) of Cre+ cells displays FS firing and expresses PV instead of SOM. It is unclear if this is off-target recombination or a real subgroup of interneurons expressing both SOM and PV at different developmental stages.

SOM neurons are highly interconnected by chemical synapses with local excitatory neurons as well as with different types of inhibitory neurons (Kapfer et al., 2007; Fino and Yuste, 2011; Levy and Reyes, 2012; Pfeffer et al., 2013; Xu et al., 2013; Jiang et al., 2015; Pala and Petersen, 2015). In addition, SOM neurons might be powerfully involved in the synchrony and oscillatory activity of the neuronal network by forming both electrical coupling within the subpopulation and chemical synapses with different neuronal subpopulations (Fanselow et al., 2008; Fanselow and Connors, 2010; Hu and Agmon, 2015; Karnani et al., 2016).

Both *in vitro* and *in vivo* electrophysiological recordings have shown that excitatory synapses onto SOM neurons are common but weaker than those formed onto PV (FS) cells (Kapfer et al., 2007; Fanselow et al., 2008; Pala and Petersen, 2015). However, the highly converging local excitatory inputs together with SOM cells membrane features such as the high input resistance, depolarized resting membrane potential and low spike threshold can powerfully recruit SOM cells into the network. Repetitive stimulation in a single pyramidal neurons is sufficient to drive a SOM cell to fire and provide feedback inhibition to pyramidal neurons *in vitro* (Kapfer et al., 2007; Silberberg and Markram, 2007) and *in vivo* (Gentet et al., 2012; Kwan and Dan, 2012). This phenomenon suggests that SOM neurons might be activated during periods of increased network activity but interestingly, *in vivo* recordings in the barrel cortex shows that activity of L2/3 SOM neurons is characterized by a high rate of discharge during quiet wakefulness and is dramatically reduced during active whisking or whisker deflections (Gentet et al., 2012). A brain state in which L2/3 SOM neurons are engaged in the network above their quiet range is unknown (Gentet et al., 2012). Optogenetic inhibition of SOM activity leads to an increase in the efficacy of excitatory connections between L2/3 pyramidal neurons (Urban-Ciecko et al., 2015) and results in the increase in burst firing of pyramidal neurons (Gentet et al., 2012). The inhibitory mechanism in this phenomenon involves the activity of both GABAA and GABAB receptors (Urban-Ciecko et al., 2015).

SOM neurons are highly active *in vivo* (Gentet et al., 2012; Pala and Petersen, 2015) and *in vitro* (Fanselow et al., 2008; Fanselow and Connors, 2010; Urban-Ciecko et al., 2015). Interestingly, SOM neurons fire persistently during UPstates and Downstates (Fanselow et al., 2008; Fanselow and Connors, 2010; Pala and Petersen, 2015; Urban-Ciecko et al., 2015) and in fact, at least in layer 2/3 their membrane potential fluctuation is lower and anticorrelated to other neurons during quiet wakefulness (Gentet et al., 2012). *In vitro*, SOM cells fire rhythmically and persistently in the theta-frequency range (3–10 Hz) in the neocortex (Fanselow and Connors, 2010) and the hippocampus (Leão et al., 2012). For this reason these cells may be involved in learning and memory processes because theta band EEG oscillations increase in power in the prefrontal cortex during working memory tasks (Krause et al., 2000).

## Input of SOM Interneurons Into Cortical Network

Somatostatin-expressing interneurons are found in all cortical layers however, they are not a uniform group (Ascoli et al., 2008; DeFelipe et al., 2013; Xu et al., 2013). In L2/3 and L5 the dominant type is the Martinotti cell, with mostly vertically directed axons, diverging in cortical L1, that target apical dendrites of pyramidal neurons (Kawaguchi and Kubota, 1997; Wang et al., 2004). SOM interneurons project densely to pyramidal cells located within a 200 μm radius (Fino and Yuste, 2011), mostly target their dendritic compartment and are recruited in a feedforward manner by activated pyramidal neurons for which they provide feedback inhibition (Fino et al., 2013). This circuit mediated by Martinotti cells modulates the effects of input arriving at apical dendrites of pyramidal neurons, thus controlling the input to supra-and infragranular layers. Also, by inhibiting generation of dendritic spike in L5 pyramids, they affect the output from the cortex (Larkum et al., 1999; Goldberg et al., 2004). The role of SOM interneurons in cortical desynchronization was examined by Chen et al. (2015), who stimulated intracortical cholinergic fibers. They found that SOM activation by acetylcholine or by optogenetics is sufficient to induce desynchronization. The study of Pfeffer et al. (2013) provided a blueprint for cortical inhibitory interactions. They recorded from Cre-mouse lines with ChR2 inserted in PV, SOM or VIP neurons and did single cell molecular profiling. The interconnectivity pattern, they found in L2/3 and L5, was that PV neurons preferentially inhibited one another, SOM inhibited intensively VIP and PV, but not SOM interneurons. VIP preferentially silenced SOM neurons. Inhibition of SOM by PV neurons was not observed. The role of SOM interneurons was also documented in another article from Scanziani Lab (Adesnik et al., 2012) that examined signal processing in visual cortex of mice with respect to mechanisms of receptive field surround suppression of pyramidal cell responses. They found that L2/3 SOM neurons are responsible for surrounding suppression and are activated preferentially by horizontal axons of L2/3 pyramids. Fu et al. (2014) explored the role of VIP-SOM circuit in mouse visual cortex in enhanced responsiveness to visual stimulation during motion and in ocular dominance plasticity. The disinhibitory VIP-SOM-pyramid circuit was found to be strongly modulated by acetylcholine acting via nicotinic receptors on VIP neurons. This circuit was recently reported to regulate plasticity in the adult brain.

SOM neurons with cell bodies in cortical L4 have axonal projections focused on FS, PV-positive interneurons and—to less extent—excitatory neurons of L4 (Xu et al., 2013). L4 SOM neurons specific function may be the release of thalamorecipient excitatory neurons from inhibition by PV-containing interneurons, which results in the opening of a gating mechanism restricting the flow of afferent information into upper cortical layers (Xu et al., 2013). In this way, SOM neurons control the output of L4.

## Involvement of Inhibitory SOM Interneurons in Brain Plasticity

The role of inhibitory interactions in neuroplastic changes has been recognized recently (Kullmann et al., 2012; Griffen and Maffei, 2014; Scheyltjens and Arckens, 2016) and in the last few years circuits involved in mechanisms of conditioning (Letzkus et al., 2011; Pi et al., 2013; Lovett-Barron et al., 2014; Wolff et al., 2014) and cortical representational plasticity (Fu et al., 2015), cortico-cortical integration (Lee et al., 2013) were identified and characterized. It is apparent that different interneuronal subtypes have a different role in controlling experience-dependent plasticity and in control of the output of principal neurons.

Robust experimental evidence exists that confirms the participation of somatostatin and somatostatin-containing interneurons in different forms of plasticity and memory formation. A positive correlation between the amount of endogenously expressed somatostatin and performance in hippocampus-dependent learning tasks has been observed in several studies (Nilsson et al., 1993; Nakagawasai et al., 2003). Intracerebroventricular administration of SOM facilitated memory (Vécsei et al., 1983, 1984; Lamirault et al., 2001) whereas its depletion impaired passive and active avoidance learning (Schettini et al., 1988; DeNoble et al., 1989), as well as water maze performance (Fitzgerald and Dokla, 1989; Guillou et al., 1998). Kluge et al. (2008) using the approach of targeted ablation of *SOM* gene observed significant reduction of long-term potentiation (LTP) in hippocampal CA1 and concluded that somatostatin appears to be indispensable for the acquisition of contextual fear memory. In line with this finding, McKay et al. (2013) observed that eye-blink conditioning results in higher intrinsic excitability of SOM interneurons and that results in enhanced inhibition onto CA1 pyramidal neurons.

Recently, the role of SOM neurons in motor training was revealed by Chen et al. (2015). They provided evidence for motor training-induced reduction in the density of cortical SOM neurons presynaptic boutons, suggesting that the resulting reduction in inhibition is essential for motor learning. In the same experiment Chen et al. (2015) found that SOM neurons were involved in regulation of dendritic spines stabilization, so activation or inhibition of SOM cells can affect both pre- and postsynaptic elements involved in plasticity. Optogenetic enhancement or suppression of SOM neurons activity in the motor cortex impaired the learning of stereotyped movements (Chen et al., 2015).

In mouse motor cortex, SOM neurons regulated the branch-specificity of dendritic Ca^2+^ spikes in L5 pyramidal neurons during motor learning and their inactivation or deletion in the primary motor cortex disturbed branch-specific dendritic calcium spikes and impaired multiple motor task learning (Cichon and Gan, 2015).

Gentet et al. (2012) and Palmer et al. (2012) considered the role of SOM neurons in gating the top-down (attentional, memory) inputs to the cortex. In a recent article, where neuronal activity during prolonged visually guided active avoidance learning was examined, Makino and Komiyama (2015) observed that the activity of L2/3 SOM neurons was reduced in the primary visual cortex of the mouse after long-lasting training, when the top-down inputs from retrosplenial cortex predominated over bottom-up visual inputs. Activation of these neurons caused the non-SOM neurons to respond in the manner they display at the beginning of the training, when the bottom-up processes dominate. The authors concluded that reduced SOM cells activity could facilitate the effects of top-down inputs, while enhanced activity promotes bottom–up inputs, and so SOM neurons act as a pathway switch. A recent article by Kato et al. (2015) demonstrated bidirectional regulation of SOM neurons activity in auditory cortex. With the use of chronic two-photon calcium imaging they found that response adaptation to the repeated tone observed in L2/3 pyramidal neurons is due to upregulation of tone-evoked responses of SOM neurons. Conversely, when the tone acquires behavioral significance, responses of SOM neurons are downregulated.

Involvement of SOM interneurons in neuroplasticty has been also demonstrated in the three most popular neuroplasticity models including ocular dominance changes, classical fear conditioning and experience-dependent barrel cortex plasticity. SOM neurons transplanted to adult visual cortex trigger the second critical period, enabling cortical plasticity and reshaping neuronal network (Tang et al., 2014). In adult visual cortex of awake head restrained mice, locomotion activates the VIP-SOM disinhibitory circuit which results in a state of facilitated, enhanced ocular dominance plasticity (Fu et al., 2015). Synaptic potentiation onto SOM neurons in amygdala is required for the expression of conditioned fear (Li et al., 2013; Penzo et al., 2014). Wolff et al. (2014) showed that UCS action in fear conditioning causes inhibition of SOM neurons in the basolateral amygdala, and that releases dendritic domains of principal neurons from inhibition and enhances integration of CS and UCS inputs. In the barrel cortex, fear conditioning with vibrissae stimulation as CS is linked to upregulation of GAD synthesis in SOM interneurons (Cybulska-Klosowicz et al., 2013).

### SOM Interneurons in Learning–Dependent Plasticity of the Barrel Cortex

The barrel cortex, which is the part of rodent primary somatosensory cortex containing representation of facial vibrissae is a widely used experimental model system for investigating cortical structure, function and plasticity (Margolis et al., 2014). Simple associative learning induces plasticity of the cortical representation of vibrissae (Siucinska and Kossut, 1996). Using [14C]-2-deoxyglucose autoradiography, we found that classical conditioning, in which the stimulation of a row of vibrissae (CS) was paired with a tail shock (UCS), resulted in an increase in the area of the barrel cortex activated by the vibrissae stimulated during conditioning (Siucinska and Kossut, 1996). In the new memory trace arising as a result of the conditioning, this enlarged cortical representation of mechanoreceptors receiving the CS may act as a memory enhancer, increasing the strength of the signal, reducing signal to noise ratio, and may facilitate the readout from memory.

At the same time, cortical representation expansion is paralleled by an extensive mobilization of GABAergic system visible in rapid increase of GAD-67 mRNA expression in L4 and increased density of GAD and GABA immunoreactive cells in the hollows of barrels of the row receiving input from the stimulated whiskers (Siucinska et al., 1999; Gierdalski et al., 2001). More inhibitory synapses appeared on spines in L4 and an increased concentration of GABA was found in the presynaptic terminals of the synapses on disynaptic spines (Jasinska et al., 2010). The physiological effect of GABA-ergic upregulation, examined by intracellular recordings, consisted in increase of frequency of spontaneous inhibitory postsynaptic currents in excitatory neurons located in barrels receiving the conditioned stimulus (CS; Tokarski et al., 2007). Investigations of GABA-ergic tonic currents revealed that they increased in excitatory L4 neurons after conditioning but markedly decreased in fast-spiking inhibitory interneurons (Urban-Ciecko et al., 2010). Reducing GABA synthesis during conditioning by intracortical injections of GAD inhibitor blocked formation of plastic change of whisker representation (Posluszny et al., 2015). The results listed above speak for modifications of inhibitory interactions in the cortex that is reorganized by the conditioning, and also for necessity of inhibition within the mechanism of learning-induced plastic change. Immunocytochemical examination of several GABA-ergic interneuron subtypes (SOM+, CR+, PV+, CB+) found an increase in the density of SOM+/GAD+ neurons in L4 of the plastic representation of the stimulated row of whiskers (Cybulska-Klosowicz et al., 2013).

Most of L4 SOM neurons belong to a different subgroup than the extensively studied Martinotti cells. SOM cells of cortical L4 have a different morphology, with the axon confined to L4 where it forms dense arborizations (Ma et al., 2006; Xu et al., 2013). They make up about 20–30% of L4 inhibitory interneurons (Rudy et al., 2011). The thalamic input to SOM neurons is weak and depressing, and the afferent sensory information to SOM cells comes through principal neurons (Beierlein et al., 2003; Cruikshank et al., 2010; Xu et al., 2013). L4 SOM interneurons can fire at much higher frequencies than those of L2/3. Xu et al. (2013) documented opposite effect of optogenetic supressing SOM cells in L2/3 than in L4 (enhancement and suppression of local excitatory neurons firing, respectively) in brain slices during UP states. It is possible that these populations of interneurons have different effects on plasticity also in awake and attentive animal. In L4, the connection probability of SOM cells with PV-expressing interneurons is higher than their connection probability with principal neurons, and they weakly inhibit principal neurons, but have strong inhibitory action upon FS (PV) neurons (Xu et al., 2013). In this way they decrease feedforward inhibition exerted by FS cells on principal neurons and disinhibit transmission of the afferent signal from the thalamus (Xu et al., 2013).

### Why is GAD Upregulated in Layer 4 SOM Neurons, and What Role can They Play During Conditioning?

**Figure 1:**
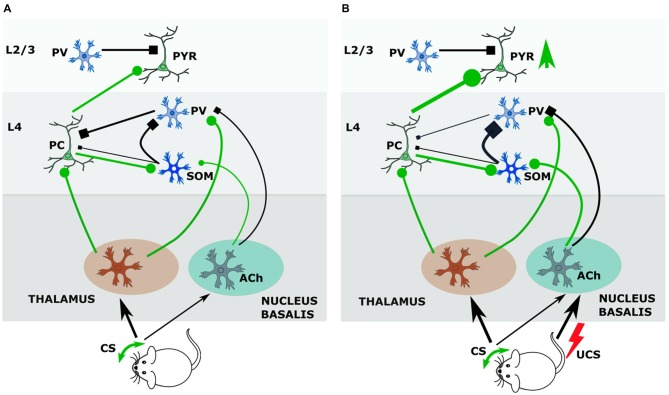
**During conditioning somatostatin (SOM) interneurons of layer 4 (L4) regulate thalamocortical input gate operated by parvalbumin (PV) interneurons. (A)** Before conditioning. During tactile stimulation of vibrissae the input from ventrobasal nucleus of thalamus targets principal cells and PV interneurons. PV interneurons by feedback inhibition control output of principal cells. Principal cells activate SOM interneurons, which inhibit PV interneurons. The input from nucleus basalis to SOM and PV neurons is weak. **(B)** Cholinergic effects during conditioning. Pairing tactile stimulus with a tail shock stimulate acetylcholine release from nucleus basalis afferents, which inhibits PV—principal cell synapse by M2 receptors, and activates SOM interneurons by nicotinic receptors. Strong activation of SOM interneurons (by principal cell and Ach) results in increased inhibition of PV interneurons and facilitates opening the thalamocortical input gate so that signal from vibrissae stimulated in conditioning achieves stronger excitation of the barrel cortex. Abbreviations: Ach, acetylcholine; CS, conditional stimulus; PC, principal cell; PV, parvalbumin-containing interneuron; PYR, pyramidal neuron; SOM, somatostatin-containing interneuron; UCS, unconditional stimulus.

We showed that the GAD upregulation was specific to mice that were conditioned and not seen in the group that received only CS, only UCS, or pseudoconditoning, so the crucial factor is the simultaneous action of CS and UCS (Siucinska and Kossut, 2006). In our experiments this lasts 0.5 s and should bring about an interaction of the CS afferent pathway with ascending neuromodulatory projections activated by the UCS. Letzkus et al. (2011) have shown that associative fear learning requires the cholinergic input to the cortex from nucleus basalis. They documented that cholinergic activation of L1 inhibitory interneurons generated inhibition of L2/3 PV inhibitory interneurons that resulted in disinhibiton of L2/3 pyramidal neurons in the sensory cortex. Since afferents from the basal forebrain also reach cortical L4 (Mesulam et al., 1983), it is plausible that in our conditioning paradigm information about aversive stimulus modifies the reception of CS in the thalamorecipient L4. The study of Cybulska-Klosowicz et al. (2013) showed that SOM neurons may contribute to this process. As described in the previous chapter, L4 SOM neurons can decrease the feedforward inhibition exerted by FS cells on principal (excitatory) neurons and thus disinhibit transmission of the afferent signal from the thalamus (Xu et al., 2013). Possibly, in response to conditioned sensory stimulus (whisker activation) and UCS (tail shock), the L4 SOM containing inhibitory network could supplement this disinhibitory effect from the L4 level, (Figure 1A) more effectively removing feed-forward inhibition of excitatory neurons during sensory input. In this way, the hypothetical mechanism of CS and UCS action upon L4 circuitry would consist in removing of gating of thalamocortical signal by PV interneurons (Figure 1B). PV interneurons inhibit, by a feedforward loop, L4 excitatory neurons, restricting their output to upper cortical layers and to SOM cells in L4. UCS-driven cholinergic projection to the cortex acts on PV cells synapses onto principal neurons via inhibitory M2 cholinergic receptors, effectively weakening the inhibition of principal cells by PV neurons (Kruglikov and Rudy, 2008). Cholinergic input to SOM interneurons, via both muscarinic and nicotinic receptors, is effective at much lower agonist doses than in other interneurons (Chen et al., 2015). Unlike PV cells, SOM neurons are activated by acetylcholine, and their activation contributes to inhibition of PV interneurons and consequently to disinhibition of principal cells. Freed from afferent inhibition by PV neurons, principal cells can in turn activate SOM cells, which would then more strongly inhibit PV neurons. This disinhibition (SOM-PV) in the thalamocortical input layer during CS+UCS pairing may allow for wider spreading of the signal from the active vibrissae in the barrel cortex (Figure 1B). Slow acting co-release of somatostatin is likely to enhance and prolong the inhibitory action of GABA released from SOM axons upon PV neuron thereby lengthening the thalamocortical gate opening period.

Activation of SOM cells during CS+UCS pairing may lead to the activity-dependent upregulation of GAD synthesis in this subtype of interneurons, resulting in the increased density of SOM+/GAD+ cells that was observed at 24-h post-training (Cybulska-Klosowicz et al., 2013). Such up-regulation of GAD synthesis by neural activity has been demonstrated previously (Welker et al., 1989; Knott et al., 2002). Somatostatin levels are also activity-regulated (Hou and Yu, 2013). These two metabolic processes may explain the increased density of SOM+/GAD+ cells observed after conditioning within the plastic cortical representation.

## Summary

Availability of long-acting agonist and use of genetically modified mice has increased our understanding of somatostatin function and its role in regulation of brain activity. However, simultaneously, a complexity of possible somatostatin effects and identification of distinct subpopulation of somatostatin-containing interneurons with characteristic electrical profile and specific anatomical features increased a diversity of somatostatinergic system activation outcome. SOM interneurons were shown to be involved in motor activity, sleep, sensory processes, cognitive functions, neuronal plasticity, while its alterations are implicated in many brain diseases like affective disorders, epilepsy or AD. Thus, despite several decades of investigations, somatostatinergic system still keeps its secrets. Regardless increasing knowledge concerning the location, distribution and action, much remains to be learned about the ways that somatostatin works and interacts with classical neurotransmitters to modulate excitability of target cells and shape the response of the neuronal networks.

## Author Contributions

ML-L, JU-C and MK carried out the research and wrote the article.

## Funding

ML-L is supported by National Science Centre, Poland (2013/09/B/NZ3/00540). JU-C is supported by National Science Centre, Poland (2015/18/E/NZ4/00721). MK is supported by National Science Centre, Poland (2015/17/B/NZ4/02016).

## Conflict of Interest Statement

The authors declare that the research was conducted in the absence of any commercial or financial relationships that could be construed as a potential conflict of interest.
